# Ticagrelor versus clopidogrel in real-world patients with ST elevation myocardial infarction: 1-year results by propensity score analysis

**DOI:** 10.1186/s12872-017-0524-3

**Published:** 2017-04-05

**Authors:** Matteo Vercellino, Federico Ariel Sànchez, Valentina Boasi, Dino Perri, Chiara Tacchi, Gioel Gabrio Secco, Stefano Cattunar, Gianfranco Pistis, Giovanni Mascelli

**Affiliations:** 1 Interventional Cardiology, Santi Antonio, Biagio e Cesare Arrigo Hospital, Alessandria, AL Italy; 2 Coronary Care Unit, Sanremo Hospital, Sanremo, IM Italy; 3 Interventional Cardiology, Sanremo Hospital, Sanremo, IM Italy; 4 Sanremo Hospital, Sanremo, IM Italy; 5 Emergency Room, Sanremo Hospital, Sanremo, IM Italy; 6 Cardiology Unit, Santi Antonio, Biagio e Cesare Arrigo Hospital, Alessandria, AL Italy; 7 Cardiology Unit, Sanremo Hospital, Sanremo, IM Italy

**Keywords:** Acute coronary syndrome, ST elevation myocardial infarction, Clopidogrel, Ticagrelor, Registry

## Abstract

**Background:**

European guidelines recommend the use of ticagrelor versus clopidogrel in patients with ST elevation myocardial infarction (STEMI). This recommendation is based on inconclusive results and subanalyses from clinical trials. Few data are available on the effects of ticagrelor in a real-world population.

**Methods:**

To compare the effects of ticagrelor and clopidogrel in a real-world STEMI population, we conducted a pre-post case-control study examining all patients with STEMI included in the Cardio-STEMI Sanremo registry between February 2011 and June 2013. Cases and controls were defined according to P2Y_12_ inhibitors, correcting the bias due to lack of randomization by propensity score analysis. Ticagrelor was introduced in 2012 in both in-hospital and pre-hospital settings independently of this study.

**Results:**

Of the 416 patients enrolled in the Cardio-STEMI registry, 401 with a definite diagnosis of STEMI were included in this study. One hundred forty-two patients received ticagrelor and 259 received clopidogrel. Regarding clinical presentation and procedural data, those in the ticagrelor group had lower CRUSADE scores (23 [14–36] vs 27 [18–38]; *p* = 0.015] but a higher proportion of radial access (33% vs 14%; *p* < 0.001), percutaneous coronary intervention (PCI; 92% vs 81 %; *p* = 0.002) and primary PCI ≤ 12 h (82% vs 66%; *p* = 0.001). The patients in the ticagrelor group had a higher procedural success rate (100% vs. 96%; *p* = 0.044). There was no difference in Bleeding Academic Research Consortium bleeding and in unadjusted incidence of hospital major adverse cardiovascular events (MACE; cardiac death, myocardial infarction, or stroke) but there was a significant reduction in unadjusted cardiac hospital death in the ticagrelor group (0.7% vs 5.4%; *p* = 0.024). After correcting for propensity score, hospital death (*p* = 0.22) and hospital MACE (*p* = 0.96) did not differ in both groups. The unadjusted survival at 1 year after STEMI was higher in the ticagrelor group (97.8% vs 87.8%; *p* = 0.024), and this result was confirmed by propensity score analysis (hazard ratio = 0.29 [0.08–0.99]; *p* = 0.048).

**Conclusions:**

In this real-word propensity score analysis, ticagrelor did not affect the risk of MACE during the hospital phase, or the incidence of hospital bleeding in patients with STEMI. However, in this mono-centric experience, ticagrelor resulted in improved 1-year survival, even after correction by propensity score.

**Electronic supplementary material:**

The online version of this article (doi:10.1186/s12872-017-0524-3) contains supplementary material, which is available to authorized users.

## Background

Patients with ST elevation myocardial infarction (STEMI) represent 32% of patients with acute coronary syndrome (ACS), with in-hospital mortality ranging from 5% to 15% according to geographic and baseline differences [1]. Each year in the United States alone, hospital costs related to acute myocardial infarction (AMI) are estimated to be as high as US$11.5 billion [2].

Dual antiplatelet therapy (DAPT) is a cornerstone of therapy for patients with STEMI. Clopidogrel has been used extensively worldwide for more than a decade; more recently, new antiplatelet agents, prasugrel and ticagrelor, have been developed and tested clinically, resulting in faster, more potent and consistent antiplatelet action [3–6].

Clopidogrel, a second-generation thienopyridine, is now available as a low-cost generic drug, with a favourable cost-effectiveness ratio. A drawback of clopidogrel is that as a pro-drug, it needs liver metabolism to be activated. In patients with STEMI, drug metabolism is hampered by specific circulatory conditions, resulting in a delayed effect of the drug compared with the time frame needed for percutaneous coronary intervention (PCI) [7, 8]. Moreover, this pathway is susceptible to genetic polymorphism, which may lead to unexpected variations in drug activity.

Ticagrelor is a novel oral, reversible P2Y12 inhibitor belonging to the cyclopentyltriazolopyrimidine class. It has a plasma half-life of 12 h. It is an active drug with more rapid onset and offset of action than clopidogrel, so that inhibition and recovery of platelet function is faster [4, 9].

A large randomized controlled trial (RCT) showed the superiority of ticagrelor compared with clopidogrel in ACS in patients with STEMI and non-ST-segment elevation myocardial infarction (NSTEMI) [6]. This large RCT led to a change in guideline recommendations in favour of ticagrelor in patients with STEMI undergoing primary PCI, largely reducing clopidogrel use in this setting. The beneficial effects of ticagrelor were demonstrated to be independent from the clinical presentation of STEMI, because no interaction has been demonstrated in the PLATO (Platelet Inhibition and Patient Outcomes) [8]. However, no specific randomized trial was designed to detect the effect of ticagrelor in patients with STEMI, leading to inconclusive and underpowered data [5, 10].

Although results from large real-world registries are important to better understand the effectiveness, use and outcomes of novel therapies [11–16], data on the benefits of ticagrelor in a real-world population of patients with STEMI are lacking. The sole large observational registry about ticagrelor in STEMI population yielded results that were in contrast with the PLATO trial, with no improvement in ischaemic events and a higher rate of bleeding for ticagrelor versus clopidogrel [17].

The safety and efficacy of drug-eluting stents (DESs) based on the generation of the device and the duration of DAPT have recently been examined. A recent meta-analysis showed that patients treated with short DAPT (<12 months) have similar survival (all-cause mortality and cardiovascular mortality) than patients treated with long DAPT (≥12 months). However, the RCTs collected in the meta-analysis examined DAPT duration, not the type of DAPT (ticagrelor vs clopidogrel), and the prevalence of ACS was widely heterogeneous (32–77%). To date, we have few data about the differences related to the type of DAPT in a population of STEMI patients treated with second-generation DESs.

We thus performed the present study to compare the effect of ticagrelor and clopidogrel in a real-world population of patients with STEMI.

## Methods

A pre-post case-control study was performed using data from the Cardio-STEMI Sanremo registry, a single-centre, ongoing, observational cohort study conducted in Sanremo Hospital, the hub for a population of 210,000 inhabitants, with 2 spokes. Every adult patient (>18 years old) admitted to the hub with a diagnosis of STEMI [18] was enrolled in the registry.

Exclusion criteria were: type 4a or 5 AMI according to the universal definition of myocardial infarction [19]; high probability of being unavailable for follow-up visits because of limited ability to cooperate or severe comorbidity with very short life expectancy.

For this study, all patients enrolled between February 2011 and June 2013 were examined. Ticagrelor became available in addition to clopidogrel in May 2012, in both in-hospital and pre-hospital settings. Since then, the choice between the two drugs has been left to the c﻿ardiologist who first makes the diagnosis of STEMI. Ticagrelor was introduced into clinical practice independently from this observational study and in accordance with European guidelines for the treatment of STEMI patients published in 2012 [20]. Patients were divided into the ticagrelor group and the clopidogrel group according to the P2Y12 inh﻿ibitor received. Local guidelines recommend the early administration of 2PY12 inhibitors, acetylsalicylic acid and unfractionated heparin immediately after a STEMI diagnosis. Therefore, in most patients presenting ﻿through﻿ the ambulance service, 2PY12 inhibitors were administered during the ambulance journey, usually after transmission of the electrocardiogram report to the hub. Patients were treated according to usual clinical practice at each institution, and PCI was performed using standard techniques. All patients received indication to continue DAPT for at least 1 year after discharge, according to the current European and American guidelines [20, 21]. Study endpoints were: (1) TIMI flow grade before and after PCI [22] and ST resolution 90 min after PCI; (2) the rate of hospital major adverse cardiovascular events (MACE; cardiovascular death, AMI and stroke) and bleeding; (3) the 12-month survival. MACE were defined according to the PLATO trial criteria, with stroke defined as focal loss of neurologic function caused by ischaemic or haemorrhagic events, with symptoms lasting at least 24 h or leading to death [6]. Myocardial infarction (MI) was defined according to the universal definition reported in the literature [19]. Bleeding Academic Research Consortium (BARC) criteria were used for bleeding assessment [23].

A paper-based case report form was prospectively collected for each patient by the referring physician. All the data were entered into an electronic database by 4 trained researchers, and the data entries were checked periodically by a data manager. The validation of in-hospital study outcomes was performed through the periodic revision of medical documents. Follow-up was performed with the support of a local health registry and a telephone interview with the patient, family physician or relatives [24]. Only mortality was inquired about during post-discharge follow-up, because this endpoint can be reliably and easily collected, and it is a surrogate for the most severe sequelae such as re-infarction, stroke or bleeding occurring during follow-up.

The Cardiology Department of Sanremo Hospital served as the data analysis centre. The study was conducted in accordance with the principles of the Helsinki Declaration and received local ethical committee approval before recruitment.

### Statistical analysis

Baseline patient and procedural characteristics are presented as means ± standard deviation, median (interquartile range), or frequency (percentage) as appropriate. The normal distribution of continuous variables in the study was determined by Shapiro-Wilks and Kolmogorov-Smirnov tests. Normally distributed continuous variables were compared using the Student t test and those with skewed distributions using the Mann-Whitney U test. Categorical variables were compared using the χ2 test or Fisher exact test. In-hospital nominal outcomes are presented as proportions and odds ratios (OR) with relative 95% confidence intervals (CI). We used Kaplan-Meier curves for survival analysis and the log-rank test to compare the treatment groups. With the aim of reducing bias related to the lack of randomization, a propensity score was calculated [25, 26] using a non-parsimonious, multivariate, binary logistic regression model.

Variables presenting a *p* value ≤0.2 for the univariate analysis and those judged to be of clinical importance, biologically plausible or supported by previously published data in the literature, were tested for inclusion in the multivariable model building process. Variables with a missing rate ≥ 5% were excluded.

Model discrimination was measured by the C statistic and calibration by the Hosmer-Lemeshow goodness-of-fit test [27]. The propensity score was used as a correction factor in a binary logistic regression to calculate the adjusted hospital outcomes and in a Cox regression analysis to examine the adjusted 1-year survival. The Cox regression results were expressed by hazard ratios (HR) with 95% CIs. All tests were two-sided. A *p* value ≤0.05 was considered statistically significant. Statistics were calculated using SPSS version 22.0 (SPSS Inc., Chicago, IL, USA).

## Results

**Fig. 1:**
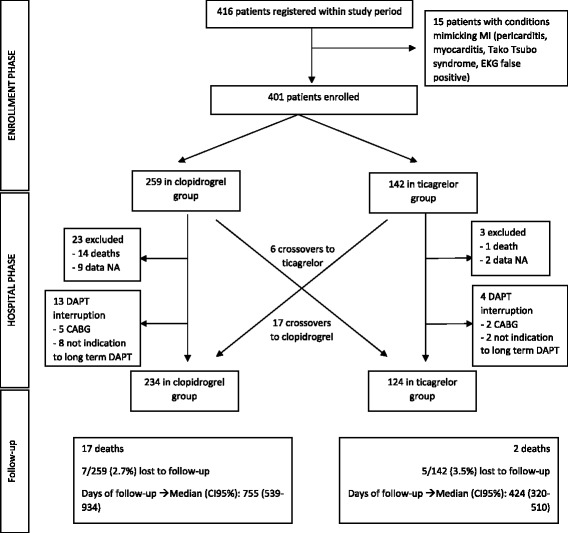
Study flowchart. CABG coronary artery bypass grafting, DAPT dual antiplatelet treatment, ECG electrocardiogram, CI confidence interval, NA not available

**Fig. 2:**
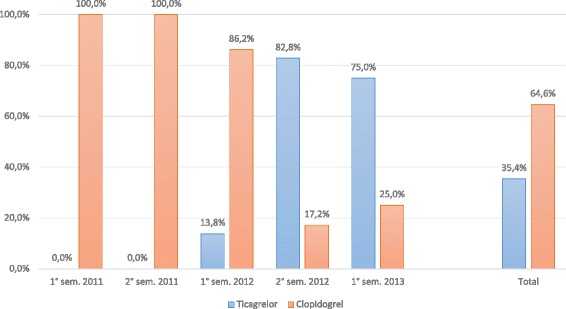
Introduction of ticagrelor in clinical practice

A total of 416 patients were enrolled in the Cardio-STEMI Sanremo registry during the study period. Fifteen patients (3.6%) were subsequently ruled out with conditions mimicking MI, and were therefore excluded from this study. The study flowchart is shown in Fig. 1. The study population included 401 patients, 259 patients in the clopidogrel group and 142 patients in the ticagrelor group. From its introduction into clinical practice in May 2012, its use had peaked at 83% by the end of 2012. Data on the adoption of ticagrelor in clinical practice are reported in Fig. 2.

Demographic and baseline data are reported in Table 1. Comparing the baseline data of the 2 groups, there were no significant differences between ticagrelor and clopidogrel, except for a lower proportion of patients aged over 75 years (21% vs 32%, respectively; *p* = 0.037), with no difference in median age.

**Table 1:** Baseline and demographic data. Data are expressed as percentage (frequency) or median (IQR)

	Ticagrelor (*n* = 142)	Clopidogrel (*n* = 259)	*p*
Age (years)	66 (56–73)	67 (56–67)	0.206
Age ≥ 75 years	21.8 (31)	32.0 (83)	*0.037*
Sex male	73.9 (105)	69.9 (81)	0.420
BMI (kg/m^2^)^a^	26 (24–30)	26 (24–29)	0.772
Hypertension	52.8 (75)	56.0 (145)	0.600
Dyslipidemia	36.6 (52)	39.0 (101)	0.668
Active smoke	45.8 (65)	37.8 (98)	0.137
Diabetes mellitus	22.5 (32)	18.5 (48)	0.362
Familiar of CAD	21.1 (30)	24.7 (64)	0.461
Previous stable angina	11.3 (16)	6.6 (17)	0.128
Previous unstable angina	3.5 (5)	4.6 (12)	0.797
Previous AMI	11.3 (16)	11.2 (29)	1.000
Previous PCI	7.0 (10)	9.3 (24)	0.574
Previous CABG	1.4 (2)	0.0 (0)	0.125
Previous CVA	4.9 (7)	7.3 (19)	0.403
PVD	16.2 (23)	11.2 (29)	0.164
Chronic kidney disease	7.0 (10)	4.6 (12)	0.361
COPD	8.5 (12)	8.9 (23)	1.000
Previous bleeding	0.7 (1)	3.9 (10)	0.106
Previous neoplasia	10.6 (15)	15.8 (41)	0.175

Italics: *p* value ≤0.05

*BMI* body mass index, *CAD* coronary artery disease, *AMI* acute myocardial infarction, *PCI* percutaneous coronary intervention, *CABG* coronary artery bypass grafting, *CVA* cerebrovascular accident, *PVD* peripheral vascular disease, *COPD* chronic obstructive pulmonary disease

^a^ *n* = 139 for ticagrelor, 258 for clopidogrel

**Fig. 3:**
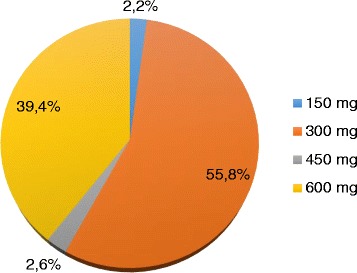
Clopidogrel loading dose

In the ticagrelor group, all patients received the loading dose, administered in 98% of cases before the cardiac catheterization laboratory; in the clopidogrel group, 236 patients (91%) received the loading dose (42% >300 mg as reported in Fig. 3), administered in 94% before the cardiac catheterization laboratory.

With regard to clinical presentation and reperfusion strategy, a lower CRUSADE score (23 [14–36] vs 27 [18–38]; *p* = 0.015], a higher rate of PCI (92% vs 80%; *p* = 0.002) and primary PCI within 12 h (82% vs 66%; *p* = 0.001) were found in the ticagrelor group compared with the clopidogrel group. (Table 2). The laboratory tests were similar between the 2 groups (Additional file 1).

**Table 2:** Clinical presentation and reperfusion strategy. Data are expressed as percentage (frequency), median (IQR), or mean (SD)

	Ticagrelor	Clopidogrel	*p*
Call to emergency service	43.0 (61/142)	41.3 (107/259)	0.75
Secondary transfer by emergency service	40.8 (58/142)	40.3 (104/258)	0.92
Hub vs spoke	67.1 (94/140)	64.5 (167/259)	0.65
Anterior AMI	46.5 (66/142)	39.8 (103/259)	0.21
New LBBB at first EKG	2.3 (5/142)	3.5 (6/256)	0.53
Cardiac frequency (bpm)	75 (65–88)	77 (63–94)	0.28
Systolic blood pressure (mmHg)	140 (120–160)	140 (120–160)	0.76
Killip class ≥ 3	5.6 (8/142)	8.1 (21/259)	0.42
Cardiac arrest	2.1 (3/142)	3.1 (8/259)	0.75
LV ejection fraction (%)	46 (40–55)	45 (40–55)	0.83
GFR Cockroft Gault (mL/min/m^2^)	72 (55–90)	67 (52–86)	0.08
GFR MDRD (mL/min/m^2^)	76 ± 25	72 ± 23	0.09
GRACE-in-hospital mortality	139 (122–157)	141 (121–167)	0.32
GRACE 6-month mortality	106 (86–128)	106 (87–125)	0.96
TIMI risk score	3 (2–5)	4 (2–5)	0.18
CRUSADE	23 (14–36)	27 (18–38)	*0.015*
Thrombolysis	1.8 (4/142)	4.6 (12/259)	0.44
Coronary angiography	100.0 (142/142)	93.1 (241/259)	*0.001*
PCI	92.3 (131/142)	80.7 (209/259)	*0.002*
Primary PCI (≤12 h after first medical contact)	82.4 (117/142)	66.4 (172/259)	*0.001*
IABP	0.7 (1/142)	3.1 (8/259)	0.17

Italics: *p* valu﻿e ≤0.05

*AMI* acute myocardial infarction, *LBBB* left bundle branch block, *EKG*, electrocardiogram, *LV* left ventricle, *GFR* glomerular filtration rate, *MDRD* modification of diet in renal disease, *GRACE* global registry of acute coronary events, *TIMI* thrombolysis in myocardial infarction, *CRUSADE* can rapid risk stratification of unstable angina patients suppress adverse outcome with early implementation of ACC/AHA Guidelines, *PCI* percutaneous coronary intervention, *IABP* intra-aortic balloon pump

Procedural data and times are reported in Table 3. The only significant difference was a higher frequency of the radial access approach for PCI in the ticagrelor group (33% vs 14%; *p* < 0.001). For patients undergoing primary PCI, the frequency of thrombus aspiration (45% vs 37%; *p* = 0.22) and the use of glycoprotein (GP) IIb/IIIa receptor antagonists (13% vs 17%; *p* = 0.41) were comparable in the ticagrelor and clopidogrel groups. With regard to the type of stent used, there were no significant differences between the clopidogrel and ticagrelor groups, although the use of bare metal stents was nominally higher in the clopidogrel group (27% vs 15%). In each group, most of the DESs were second-generation devices.

**Table 3:** Procedural data in patients with STEMI and in primary PCI. Data are expressed as percentage (frequency) or median (IQR)

	Ticagrelor	Clopidogrel	*p*
Radial vs femoral	33.1 (47/142)	14.5 (35/241)	*<0.001*
Left main	1.4 (2/142)	2.1 (5/241)	1.00
Multi-vessel CAD	36.6 (52/142)	36.5 (88/241)	1.00
Culprit vessel			0.19
RCA	38.7 (53/137)	38.9 (91/234)	
CX	10.9 (15/137)	17.9 (42/234)	
LAD	48.9 (67/137)	41.5 (97/234)	
Other	1.5 (2/137)	1.7 (4/234)	
Complete revascularization	58.0 (76/131)	56.9 (119/234)	0.91
Total contrast amount (mL)	200 (147–268)	203 (148–284)	0.89
Primary PCI			
Multi-vessel CAD	35.9 (42/117)	34.9 (88/241)	0.90
Culprit vessel			0.44
RCA	38.8 (45/116)	39.0 (67/172)	
CX	11.2 (13/116)	15.7 (27/172)	
LAD	48.2 (56/116)	43.0 (74/172)	
Others	1.8 (2/116)	2.3 (4/172)	
TIMI flow pre-PCI	1 (0–3)	1 (0–3)	0.09
Reperfusion before PCI (TIMI 3)	26.5 (31/117)	16.9 (29/172)	*0.047*
Reperfusion before PCI (TIMI 2 o 3)	45.3 (53/117)	37.8 (65/172)	0.22
Type B2-C ACC/AHA lesion classification	73.6 (81/110)	74.5 (117/155)	0.78
Thrombus aspiration	44.8 (52/116)	37.2 (64/172)	0.22
GPIIb/IIIa receptor antagonist	12.8 (15/117)	16.9 (29/172)	0.41
Stent type			0.15
BMS	14.7 (17/116)	27.1 (46/170)	
First-generation DES	0.9 (1/116)	0.6 (1/170)	
Second-generation DES	75.0 (86/116)	61.8 (104/170)	
BMS and DES	2.6 (3/116)	2.4 (4/170)	
No stent	7.8 (9/116)	8.8 (15/170)	
Symptom onset to EKG (hh:min)	1:25 (0:40–2:26)	1:19 (0:50–2:41)	0.84
EKG to door hub (hh:min)	1:26 (1:14–2:10)	1:21 (1:06–1:51)	0.25
Transfer time (hh:min)	0:42 (0:36–0:52)	0:38 (0:34–0:48)	0.11
DTB min (hh:min)	1:46 (1:35–2:37)	1:49 (1:34–2:30)	0.75
DTB ≤60 min	3.5 (4/114)	3.0 (5/166)	1.00
DTB ≤90 min	20.2 (23/114)	21.7 (36/166)	0.88
DTB ≤120 min	52.6 (60/114)	52.4 (87/166)	1.00
Total ischaemic time(hh:min)	3:50 (2:45–5:34)	3:30 (2:42–5:39)	0.37

I﻿tali﻿cs: *p* value ≤0.0﻿5

*CAD* coronary artery disease, *RCA* right coronary artery, *CX* circumflex coronary artery, *LAD* left anterior descending, *ACC/AHA* American College of Cardiology/American Heart Association, *GPIIb/IIIa* glycoprotein IIb/IIIa, *PCI* percutaneous coronary intervention, *TIMI* thrombolysis in myocardial infarction, *BMS* bare metal stent, *DES* drug-eluting stent, *EKG* electrocardiogram, DTB door to balloon

The system-related delay, patient-related delay and the time intervals were not different between the 2 groups.

Discharge therapy was not significantly different between the ticagrelor and clopidogrel groups (Additional file 2).

Procedural success, defined either as TIMI 3 or TIMI 2–3 with stenosis <50% after primary PCI, was higher in the ticagrelor group than in the clopidogrel group (99% vs 90%, *p* = 0.001; 100.0% vs. 96%, *p* = 0.044), whereas the proportion of patients with ≥50% ST resolution (78% vs 78%; *p* = 0.879) or ≥70% ST resolution (60% vs 67%; *p* = 0.294) was similar between the groups. Data on procedural outcomes are reported in Table 4.

**Table 4:** Procedural and hospital outcomes. Data are expressed as percentage (frequency) or median (IQR)

	Ticagrelor (*n* = 142)	Clopidogrel (*n* = 259)	*P*
Procedural outcomes (unadjusted analysis)			
TIMI flow post-PCI	3 (3–3)	3 (3–3)	*0.002*
Procedural success (TIMI 3 and stenosis <50%)	99.0 (115/116)	89.9 (151/168)	*0.001*
Procedural success (TIMI 2–3 and stenosis <50%)	100.0 (116/116)	95.8 (161/168)	*0.044*
STR%	89 (50–100)	100 (50–100)	0.482
STR ≥ 50%	78.1 (82/105)	78.2 (122/156)	0.879
STR% ≥ 70%	60.0 (63/105)	66.7 (104/156)	0.294
Hospital outcomes (unadjusted analysis)			
In-hospital all-cause death	0.7 (1)	5.4 (14)	*0.024*
In-hospital CV death	0.7 (1)	5.4 (14)	*0.024*
In-hospital non-fatal AMI	3.5 (5)	1.2 (3)	0.14
Ischaemic CVA	0.4 (1)	0.7 (1)	1.00
Total CVA (ischaemic or haemorrhagic)	0.4 (1)	0.7 (1)	1.00
Hospital MACE	4.9 (7)	6.9 (18)	0.52
Definite stent thrombosis	1.4 (2)	0.8 (2)	0.62
Final LVEF [%]	50 (40–55)	48 (40–55)	0.55
CPK peak [UI/L]	1347 (535–2590)	1376 (677–2491)	0.77
Hospital stay [days]	4 (4–6)	5 (4–6)	0.84

Italics﻿: *p* value ≤0.05

*TIMI* thrombolysis in myocardial infarction, *STR* ST resolution, *CV* cardiovascular, *AMI* acute myocardial infarction, *CVA* cerebrovascular accidents, *LVEF* left ventricular ejection fraction, *CPK* creatine phosphokinase

In the unadjusted analysis, there was no difference in hospital MACE (cardiovascular death, non-fatal MI, stroke) between the ticagrelor and clopidogrel groups (4.9% vs 6.9%; *p* = 0.520; OR, 0.69 [95% CI, 0.28–1.70]). However, the use of ticagrelor resulted in a significant reduction of cardiovascular mortality (0.7% vs 5.4%; *p* = 0.024; OR, 0.12 [95% CI, 0.02–0.95]). No difference was found in new hospital non-fatal AMI (3.5% vs 1.2%, *p* = 0.14) or in cerebrovascular accidents (0.4% vs 0.7% vs 0.8%; *p* = 1.000) (Table 4). No significant difference between the ticagrelor and clopidogrel groups was found in stent thrombosis (1.4% vs 0.8%, *p* = 0.62) and infarct size, estimated by the peak creatine kinase value (1347 vs 1372 UI/L, *p* = 0.77), or for left ventricular ejection fraction (50% vs 48%, *p* = 0.55) before hospital discharge. The causes of cardiovascular death are reported in Additional file 3.

BARC bleeding was similar between the 2 groups; there was no difference in BARC categories for BARC ≥2 and BARC ≥3. The lowest median haemoglobin value (12.2 vs 12.2 g/dL; *p* = 0.8) and the difference between the highest and lowest haemoglobin value during hospital stay (2.2 vs 2.1 g/dL; *p* = 0.9) were similar between the ticagrelor and clopidogrel groups. The risk of transfusion and the amount of packed red blood cell units did not show any significant difference (Additional file 4).

**Fig. 4:**
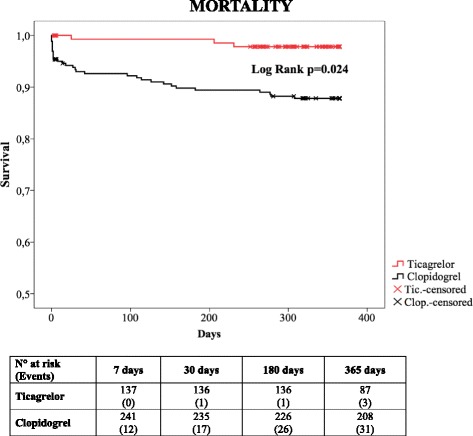
Kaplan-Meier survival analysis for ticagrelor (*red*) vs clopidogrel (*black*) for 1 year of follow- up

In the Kaplan-Meier analysis, unadjusted 1-year survival probability was higher in the ticagrelor group than in the clopidogrel group (97.8% vs 87.8%; log-rank *p* = 0.024) (Fig. 4), as was the probability of 1-year survival free from cardiovascular death (97.8% vs 90.2%; log-rank *p* = 0.005) (Additional file 5).

### Propensity score analysis

A total of 21 interfering variables were included in the propensity score and are reported in Additional file 6. Candidate variables included sex, age, BMI, smoking, diabetes, dyslipidemia, familiar history of coronary artery disease, cerebrovascular accident (CVA), bleeding, previous AMI or PCI, glomerular filtration rate, TIMI risk score, CRUSADE score, anterior AMI, first medical contact through the ambulance service, admission to hub vs spoke, left ventricular ejection fraction, Killip class III-IV, radial access, primary PCI ≤12 h.

**Fig. 5:**
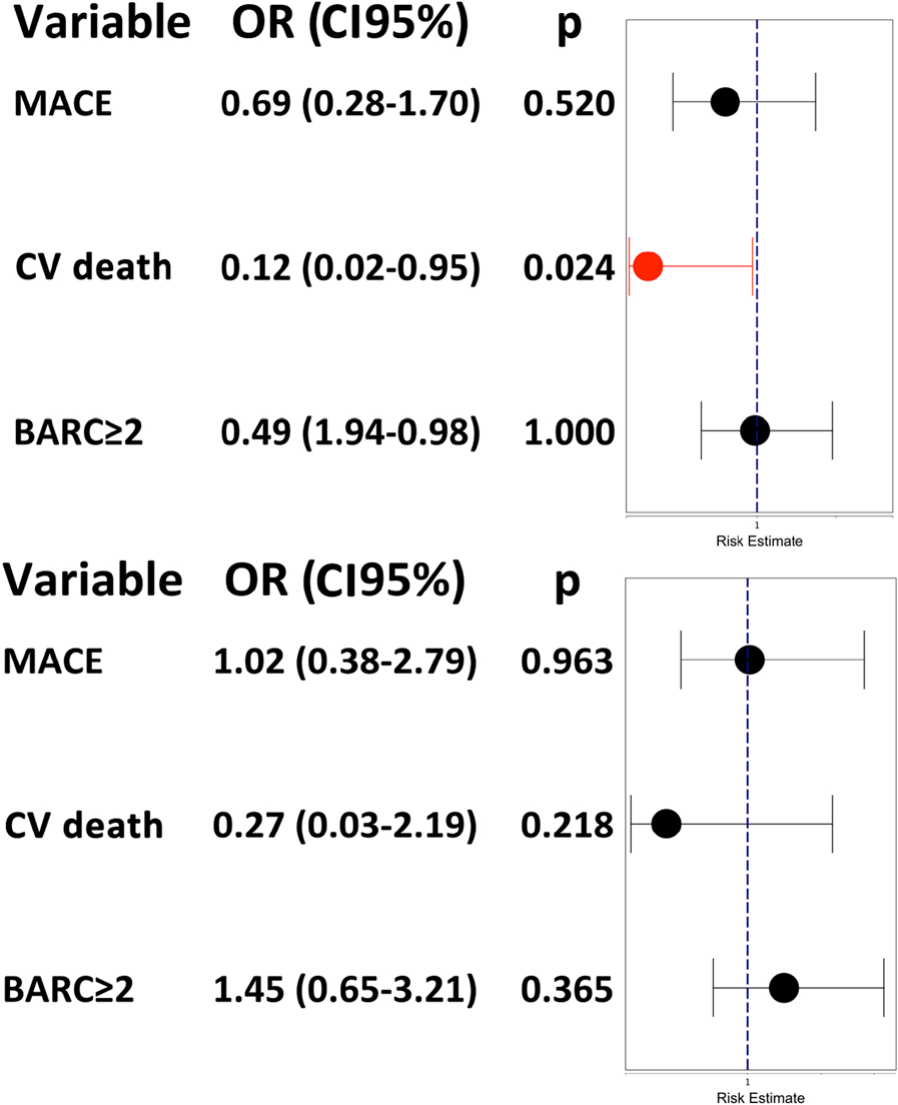
Forest plot for in-hospital major adverse cardiovascular events (MACE), cardiovascular death and Bleeding Academic Research Consortium (BARC) bleeding. Unadjusted (*at the top*) and propensity-adjusted (*at the bottom*) odds ratio (OR) for in-hospital MACE, cardiovascular death and BARC bleedings ≥2. CI confidence interval

The propensity score was calculated for 368 patients (missing for 33, 8.2%) and showed good discrimination (area under the curve =0.695 [0.640–0.751]; *p* < 0.01) and good calibration (*p* = 0.355). Using propensity score regression, there were no differences in mortality, ischaemic or haemorrhagic events during the hospital stay. As showed in Fig. 5, after adjusting for the propensity score, hospital cardiovascular mortality (OR, 0.27 [95% CI, 0.03–2.19]; *p* = 0.218) and hospital MACEs (OR, 1.02 [95% CI, 0.38–2.79];], *p* = 0.963) were similar in the ticagrelor and clopidogrel groups.

However, the survival analysis adjusted by propensity score demonstrated that all-cause mortality at 1 year after STEMI remained significantly lower for the ticagrelor group (HR, 0.29 [0.08–0.99]; *p* = 0.048).

There were 6 crossovers from clopidogrel to ticagrelor (3 for STEMI occurring on clopidogrel therapy, 3 for unknown cause), and 17 crossovers from ticagrelor to clopidogrel (4 for high bleeding risk or vitamin K antagonist [VKA], 2 for dyspnoea, 2 for low patient compliance, 4 for low ischaemic risk after coronary angiography, 4 for unknown cause). Seven of 401 patients were proposed for elective coronary artery bypass graft (1.7%) during hospitalization, 5 in the clopidogrel group and 2 in the ticagrelor group. Ten patients were discharged without DAPT for high bleeding risk or contemporary VKA therapy (Fig. 1).

## Discussion

In real-world populations of patients with STEMI admitted to an Italian hospital, treatment with ticagrelor reduced the mortality rate at 1 year compared with clopidogrel, with a risk of mortality 3.4 times higher for clopidogrel than for ticagrelor. This was confirmed when propensity analysis was used to reduce the risk of bias because of lack of randomization. Ticagrelor did not reduce hospital MACE after propensity score correction, and it did not affect hospital bleeding according to the BARC classification.

The new 2PY12 inhibitors, ticagrelor and prasugrel, are known to have more favourable pharmacologic proprieties for ACS than clopidogrel. Because of more predictable pharmacokinetics and a more potent and constant effect with faster onset, there is strong rationale for their use in patients with STEMI [4, 9]. However, despite promising pharmacological data and European guidelines recommendations to administer the new 2PY12 inhibitors in patients with STEMI [28], there is still uncertainty about their real clinical efficacy compared with the older and more widely adopted clopidogrel.

The STEMI population differs from the larger ACS population in several respects, because it is composed of younger patients with less comorbidities and lower procedural haemorrhage risk, but higher incidence of hemodynamic instability at presentation. Moreover, conditions affecting drug bioavailability, such as reduced gastrointestinal absorption, vomiting and morphine administration, can reduce the effect of P2Y12 more in patients with STEMI than other ACS [8, 29]. In an RCT directly comparing ticagrelor and clopidogrel, patients with STEMI had a delayed onset of both these P2Y12 inhibitors [30]. A delayed onset of action for ticagrelor was also confirmed in a small randomized trial measuring platelet reactivity after a loading dose of ticagrelor and prasugrel in patients with STEMI [9].

With no randomized trial focusing on the effect of ticagrelor or prasugrel versus clopidogrel in a STEMI population and the uncertainties about pharmacokinetics, the source of evidence on the new 2PY12 inhibitors in the STEMI population is still the subgroup analysis in the PLATO and TRITON-TIMI 38 trials [10].

The TRITON-TIMI trial subgroup analysis showed that prasugrel was superior to clopidogrel (300 mg loading dose/75 mg maintenance dose) in 3534 patients with STEMI undergoing primary or secondary PCI when considering cardiovascular death, non-fatal MI, or non-fatal stroke at 30 days (115 [6.5%] vs 166 [9.5%]; 0.68 [0.54–0.87]; *p* = 0.0017] and at 15 months (174 [10.0%] vs 216 [12.4%]; 0.79 [0.65–0.97]; *p* = 0.0221). A subanalysis in the PLATO trial on 7544 patients with STEMI has shown no superiority in MACE (cardiovascular death, non-fatal MI and stroke) for ticagrelor versus clopidogrel at 12 months follow-up, although there was a nominal trend in favour of ticagrelor (HR, 0.87 [0.75–1.01]; *p* = 0.07). However, in this analysis, ticagrelor significantly reduced secondary endpoints such as total mortality (HR 0.82; *p* = 0.05), MI (HR, 0.8; *p* = 0.03), and definite stent thrombosis (HR 0.66; *p* = 0.03).

Because the PLATO and TRITON-TIMI 38 trials are heterogeneous with regard to patient characteristics and treatments, their results could not be compared. The TRITON-TIMI 38 trial enrolled clopidogrel-naive patients undergoing primary and secondary PCI, whereas in PLATO almost 50% of patients were preloaded with open-label clopidogrel (300 or 600 mg), reducing the benefit of ticagrelor in the early phase of follow-up. Considering the PLATO subgroup analysis, ticagrelor did not reduce the primary endpoint at 12 months but conferred a survival advantage after 30 days, and this may be the result of a mechanism that differs from antiplatelet activity. Type 1 equilibrative nucleoside transporter (ENT1) protects adenosine from intercellular metabolism. Inhibiting ENT1 increases the concentration and biological activity of adenosine, particularly at sites of ischemia and tissue injury where it is formed [31]. Furthermore, in patients with STEMI, the beneficial effect of ticagrelor may be more pronounced in a subpopulation of high-risk patients characterized by high on-treatment platelet reactivity even after large loading doses of clopidogrel [32]. In these patients, even a double dose of clopidogrel did not reduce cardiovascular events compared with the standard dose, as demonstrated by the CURRENT-OASIS trial [33].

Despite the large number of patients included, PLATO and TRITON-TIMI 38 were not designed specifically to assess the effect of P2Y12 in patients with STEMI, and both trials are underpowered to reach a definitive conclusion; the low quality of this conclusion is summarized in the B level of evidence in the European guidelines [28].

In the absence of high-quality RCTs, however, there is increasing interest in prospective observational registries. The Swiss ATACS registry analysed the effect of prasugrel on a STEMI population, demonstrating that it is advantageous over clopidogrel (in-hospital mortality 1.7% vs 4.4%) at the expense of an increased bleeding risk (significantly superior for prasugrel on adjusted but not crude analysis) [15]. Another Swiss observational study, the Swiss ACS bleeding score, demonstrated that clopidogrel and prasugrel had similar safety profiles at 30 days (BARC 3, 4, 5 adjusted HR, 0.75 [0.42–136]) and at 1 year (BARC 3, 4, 5 adjusted HR, 0.67 [0.38–1.20]), without considering efficacy [14]. The SCAAR registry from Sweden demonstrated that ticagrelor increased survival and reduced bleeding risk in selected patients with ACS at low risk of bleeding [11]. Conversely, there is no European registry on the effect of ticagrelor in the STEMI population. The Greek GRAPE registry considered the clinical effect of clopidogrel, prasugrel, and ticagrelor in the general ACS population, hindering conclusions on the STEMI population. The GRAPE study demonstrated that ticagrelor does not reduce the rate of MACE at 1 year (HR, 0.78 [0.54–1.12]), whereas the bleeding rate increased (HR, 1.81 [1.55–2.10]) [16].

Our study is one of the few available registries on the effect of ticagrelor in a STEMI population and is the first to focus on the European setting. It has confirmed the findings from the PLATO trial. Our study demonstrated no benefit for ticagrelor with regard to cardiovascular death, AMI and stroke during the hospital phase. The risk reduction observed for in-hospital unadjusted cardiovascular mortality was not confirmed after propensity score analysis. This may be because of the use of a high clopidogrel loading dose (over 42% patients ≥300 mg) in the pre-hospital phase through the ambulance service. These results are concordant with the PLATO trial, where the beneficial effect of ticagrelor on MACE was achieved only after 30 days.

In our data, ticagrelor did not increase the risk of bleeding according to the BARC classification. This may depend on the lower haemorrhagic risk of the STEMI population compared with the general ACS population. The low haemorrhagic risk could have reduced the rate of clinically relevant bleeding even if the drug demonstrated a larger antiplatelet effect. Both these findings are in accordance with the PLATO subanalysis on STEMI.

A recent meta-analysis found a 20% reduced rate of MACE in patients on the new P2Y12 compared with clopidogrel [34], at the expense of a 50% increase in the risk of stroke. This was not confirmed in our study, where the proportions of MACE and stroke were similar in the 2 groups.

The main result of the present study, a reduction in 1-year mortality for ticagrelor, is concordant with the results of the PLATO study and the substudy in the STEMI population. In our registry, limited by the small sample size, this finding may be the result of statistical chance; however, it may also result from pharmacodynamic proprieties of ticagrelor that are distinct from its antiplatelet function, such as adenosine mimetic action [31]. Moreover, it may result from a favourable ratio between the unavoidable haemorrhagic risk and protection from ischaemic events. The observational registry by Park et al. [17] on ticagrelor in a STEMI population yielded contrasting results, showing that it did not reduce the risk of ischaemic events but was associated with an increased risk of bleeding compared with clopidogrel after controlling for propensity score. That study was conducted in Korea, thus it may lack external validity in Europe due to genetic variations and difference in patient characteristics, such as BMI or age. Furthermore, Park et al. considered only patients with STEMI undergoing PCI, excluding patients with STEMI undergoing medical therapy, where the beneficial effect of ticagrelor vs clopidogrel is increased [35].

In the present study, there was a 10% improvement in the success rate of primary PCI in patients on ticagrelor, with improvement in post-PCI TIMI 3 score (*p* = 0.001). This result came from crude data, without randomization or propensity score correction. Therefore, the better angiographic results in patients treated with ticagrelor could be secondary to chance. The PLATO angiographic substudy [36] showed that neither coronary flow nor myocardial perfusion demonstrated a difference with ticagrelor versus clopidogrel. However, in this substudy, the time interval between randomization and angiography or PCI was really short, particularly in the STEMI patients. In our institution, many patients were pretreated before arrival at the catheterization laboratory and the time for the pharmacological effect was longer. This fact, associated with greater platelet inhibition with ticagrelor, could have improved angiographic outcomes. Ticagrelor is also known to inhibit cell uptake of adenosine [31], and it could also be hypothesized that ticagrelor might increase the concentration of adenosine in the myocardium more than clopidogrel, inducing hyperemia and vasodilation, which is inconsistent with the results of the PLATO angiographic subgroup, where there was no improvement in coronary flow after percutaneous revascularization [36]. We should underline that this finding came from crude analysis and it could be biased by the lack of randomization or propensity score correction. However, in our sample, as in the PLATO angiographic subgroup, ticagrelor and clopidogrel achieved a similar proportion of ST resolution after PCI, with no effects on cardiac reperfusion.

Ticagrelor was discontinued in 2% of patients due to dyspnoea, a previously reported complication [6]. Other patients were converted to clopidogrel because of compliance problems, increased haemorrhagic risk or contemporary VKA treatment. There was only one intra-hospital death in the ticagrelor group (caused by cardiogenic shock), compared with 14 intra-hospital deaths in the clopidogrel group; this difference is relevant but it was not significant after controlling for the propensity score.

### Limitations

This study has the limitations of a prospective case-control study. There were some differences between baseline and procedural data. Cases and controls were enrolled in different time periods, and this may contribute to confounding and to the different sizes of the study groups. However, we used propensity scores to minimize the bias related to lack of randomization, a strategy commonly used in other studies based on similar prospective registries. Power may be limited by sample size, however to our knowledge this study is the largest real-world registry on ticagrelor in Europe, even though the study was not powered for hard endpoints. The use of radial access was lower than the current standard and this issue could have had an unfavorable impact, increasing the bleeding risk in our sample. However, the type of access was included in the propensity score model in order to reduce the interference related to the differences between ticagrelor and clopidogrel groups. One further limitation is the lack of data collection on MACE and bleeding after the hospital phase. This may have influenced the perception of the favourable effects on the rate of MACE, which may become apparent only after 30 days [6]. However, this choice is often used during follow-up data collection within observational registries, as mortality is more easily and reliably collected after discharge, and it may be considered a surrogate outcome of complications including MACE and bleeding. Although the 2012 ESC guidelines recommended the use of ticagrelor or prasugrel in place of clopidogrel, in our institution prasugrel was not introduced during the time window examined in this study. This is mainly because prasugrel should not be administered in patients older than 75 years, with low body weight (≤60 kg) or with a history of previous CVA. So its introduction in our STEMI network, particularly in the pre-hospital setting, was considered difficult. We have no data on the duration of DAPT; however, each patient received indication to continue DAPT for at least 12 months after STEMI.

## Conclusions

In this real-world single-centre experience, ticagrelor resulted in improved survival at 1 year versus clopidogrel in patients with STEMI, even after propensity score correction. Ticagrelor did not reduce the composite outcome of in hospital MACE, and it did not increase the risk of in-hospital bleeding. Although these results confirm data from previous subanalysis in STEMI patients, large RCTs are warranted to confirm the positive effect of ticagrelor shown in this population.

## Additional files

Additional file 1: Baseline laboratory data. (DOCX 15 kb)

Additional file 2: Discharge therapy. Data are expressed as percentage (frequency). (DOCX 11 kb)

Additional file 3: Intra-hospital causes of death. Data are expressed as percentage (frequency). (DOCX 11 kb)

Additional file 4: Data on BARC bleeding. Data are expressed as percentage (frequency) or median (IQR). (DOCX 12 kb)

Additional file 5: Unadjusted Kaplan-Maier analysis on cardiovascular mortality at 1 year. (DOCX 51 kb)

Additional file 6: Interfering variables retained in the propensity score model. (DOCX 11 kb)

## Abbreviations

ACS: Acute coronary syndrome
AMI: Acute myocardial infarction
BARC: Bleeding Academic Research Consortium
BMI: Body mass index
CI: Confidence interval
CRUSADE: Can rapid Risk stratification of Unstable angina patients Suppress ADverse outcome with Early implementation of ACC/AHA Guidelines
CVA: Cerebrovascular accident
DAPT: Dual antiplatelet treatment
DES: Drug-eluting stent
HR: Hazard ratio
IQR: Interquartile range
MACE: Major adverse cardiovascular event
MI: Myocardial infarction
NSTEMI: Non-ST-segment elevation myocardial infarction
OR: Odds ratio
PCI: Percutaneous coronary intervention
RCT: Randomized controlled trial
STEMI: ST-segment elevation myocardial infarction
TIMI score: Thrombolysis in myocardial infarction score
VKA: Vtamin K antagonist
