# Supplementary material for: Ticagrelor versus clopidogrel in real-world patients with ST elevation myocardial infarction: 1-year results by propensity score analysis
Source: BMC Cardiovasc Disord. 2017 Apr 5;17:97. doi: 10.1186/s12872-017-0524-3 (PMC5382425; doi:10.1186/s12872-017-0524-3)
Supplement: Supplementary file 1 — Baseline laboratory data. (DOCX 15 kb) [file 12872_2017_524_MOESM1_ESM.docx]

**Additional file 1**

**Baseline laboratory data**

|  | **Ticagrelor** | **Clopidogrel** |  |
| --- | --- | --- | --- |
| Glycaemia (mg/dL) | 149 (124–187) | 144 (120–193) | 0.64 |
| Glycated haemoglobin (%) | 5.9 (5.5–6.7) | 5.9 (5.7–6.5) | 0.32 |
| White blood cells (×10^3^ μL) | 9.8 (7.8–12.0) | 10.2 (8.0–12.9) | 0.23 |
| Platelets (×10^3^ μL) | 229 (195–274) | 227 (191–267) | 0.40 |
| Haemoglobin (g/dL) | 14.5 (13.0–15.5) | 13.3 (14.4–15.6) | 0.98 |
| Cholesterol (mg/dL) | 177 (153–209) | 176 (154–207) | 0.56 |
| HDL (mg/dL) | 44 (37–51) | 41 (33–52) | 0.07 |
| Triglycerides (mg/dL) | 109 (77–149) | 108 (82–154) | 0.58 |
| LDL (mg/dL) | 108 (83–129) | 109 (86–132) | 0.38 |

*HDL* high density lipoprotein, *LDL* low density lipoprotein
