# Supplementary material for: Ticagrelor versus clopidogrel in real-world patients with ST elevation myocardial infarction: 1-year results by propensity score analysis
Source: BMC Cardiovasc Disord. 2017 Apr 5;17:97. doi: 10.1186/s12872-017-0524-3 (PMC5382425; doi:10.1186/s12872-017-0524-3)
Supplement: Supplementary file 2 — Discharge therapy. Data are expressed as percentage (frequency). (DOCX 11 kb) [file 12872_2017_524_MOESM2_ESM.docx]

**Additional file 2**

**Discharge therapy. Data are expressed as percentage (frequency)**

|  | **Ticagrelor (140)** | **Clopidogrel (241)** | **p** |
| --- | --- | --- | --- |
| Statins | 92.1 (129) | 90.9 (219) | 0.67 |
| β-Blockers | 71.4 (100) | 70.5 (170) | 0.85 |
| ACE inhibitors or ARBs | 61.4 (86) | 62.2 (150) | 0.88 |
| Diuretics | 25.7 (36) | 29.5 (71) | 0.43 |

*ACE* angiotensin converting enzyme, *ARB* angiotensin receptor blocker
