# Supplementary material for: Ticagrelor versus clopidogrel in real-world patients with ST elevation myocardial infarction: 1-year results by propensity score analysis
Source: BMC Cardiovasc Disord. 2017 Apr 5;17:97. doi: 10.1186/s12872-017-0524-3 (PMC5382425; doi:10.1186/s12872-017-0524-3)
Supplement: Supplementary file 3 — Intra-hospital causes of death. Data are expressed as percentage (frequency). (DOCX 11 kb) [file 12872_2017_524_MOESM3_ESM.docx]

**Additional file 3**

**Intra hospital causes of death. Data are expressed as percentage (frequency)**

|  | **Ticagrelor (n=142)** | **Clopidogrel (n=259)** |
| --- | --- | --- |
| Cardiogenic shock | 0.7 (1) | 1.9 (5) |
| Pulseless electrical activity | – | 1.9 (5) |
| Asystole | – | 0.7 (2) |
| Cardiac rupture or tamponade | – | 0.7 (2) |
