# Supplementary material for: Ticagrelor versus clopidogrel in real-world patients with ST elevation myocardial infarction: 1-year results by propensity score analysis
Source: BMC Cardiovasc Disord. 2017 Apr 5;17:97. doi: 10.1186/s12872-017-0524-3 (PMC5382425; doi:10.1186/s12872-017-0524-3)
Supplement: Supplementary file 4 — Data on BARC bleeding. Data are expressed as percentage (frequency) or median (IQR). (DOCX 12 kb) [file 12872_2017_524_MOESM4_ESM.docx]

**Additional file 4**

**Data on BARC bleedings. Data are expressed as percentage (frequency) or median (IQR)**

|  | **Ticagrelor (n=142)** | **Clopidogrel (n=259)** | **p** |
| --- | --- | --- | --- |
| BARC type 2 | 6.3 (9) | 4.6 (12) | 0.487 |
| BARC type 3 | 3.5 (5) | 4.2 (11) | 0.796 |
| 3A | 2.8 (4) | 2.7 (7) | 1.000 |
| 3B | 0.7 (1) | 1.5 (4) | 0.660 |
| BARC type 4 | 0.0 | 0.0 |  |
| BARC type 5 | 0.0 | 1.2 (3) | 0.555 |
| 5A | 0.0 | 0.0 |  |
| 5B | 0.0 | 1.2 (3) | 0.555 |
| BARC≥2 | 9.9 (14) | 10.0 (26) | 1.000 |
| BARC≥3 | 3.5 (5) | 5.4 (14) | 0.469 |
| Lowest haemoglobin level (g/dL) | 12.2 (11.2–13.3) | 12.2 (10.9–13.4) | 0.881 |
| Delta haemoglobin (g/dL) | 2.2 (1.3–3.0) | 2.1 (1.2–2.9) | 0.934 |
| Delta haemoglobin categories |  |  | 0.776 |
| <3 g/dL | 74.5 (105) | 75.1 (190) |  |
| 3–5 g/dL | 24.1 (34) | 22.5 (57) |  |
| ≥5 g/dL | 1.4 (2) | 2.4 (6) |  |
| Transfusion | 2.1 (3) | 3.1 (8) | 0.753 |
| Transfusion (IU) | 2.0 (2.0–2.75) | 2 (2–2.5) | 0.630 |
