# Supplementary material for: Ticagrelor versus clopidogrel in real-world patients with ST elevation myocardial infarction: 1-year results by propensity score analysis
Source: BMC Cardiovasc Disord. 2017 Apr 5;17:97. doi: 10.1186/s12872-017-0524-3 (PMC5382425; doi:10.1186/s12872-017-0524-3)
Supplement: Supplementary file 5 — Unadjusted Kaplan-Maier analysis on cardiovascular mortality at 1 year. (DOCX 51 kb) [file 12872_2017_524_MOESM5_ESM.docx]

**Additional file 5**

**Unadjusted Kaplan-Maier analysis on cardiovascular mortality at 1 year**

**
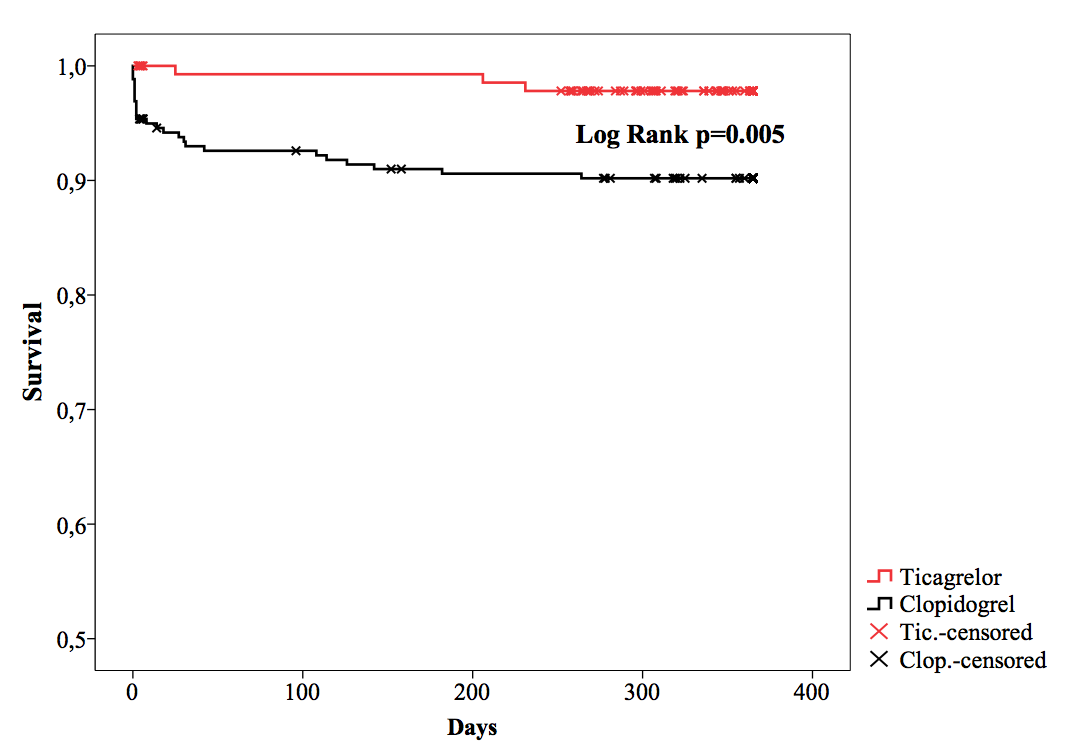
**

|  | **No. at risk (events)** | | | |
| --- | --- | --- | --- | --- |
|  | **7 days** | **30 days** | **180 days** | **365 days** |
| **Ticagrelor** | 137 (0) | 136 (1) | 136 (1) | 87 (3) |
| **Clopidogrel** | 241 (12) | 235 (17) | 226 (23) | 208 (25) |
