# Supplementary material for: Ticagrelor versus clopidogrel in real-world patients with ST elevation myocardial infarction: 1-year results by propensity score analysis
Source: BMC Cardiovasc Disord. 2017 Apr 5;17:97. doi: 10.1186/s12872-017-0524-3 (PMC5382425; doi:10.1186/s12872-017-0524-3)
Supplement: Supplementary file 6 — Interfering variables retained in the propensity score model. (DOCX 11 kb) [file 12872_2017_524_MOESM6_ESM.docx]

**Additional file 6**

**Interfering variables retained in the propensity score model**

| **Baseline characteristics** | **Procedural data** |
| --- | --- |
| Sex | TIMI risk score |
| Age | CRUSADE |
| BMI (kg/m^2^) | Anterior AMI |
| Active smoker | Admission through ambulance service |
| Diabetes mellitus | Hub admission |
| Dyslipidaemia | LVEF |
| Familial history of CAD | Killip class≥3 |
| Previous CVA | Radial access |
| Previous hemorrhage | p-PCI≤12 h |
| Previous AMI |  |
| Previous PCI |  |
| GFR MDRD (ml/min/m^2^) |  |

*TIMI* thrombolysis in myocardial infarction, *BMI* body mass index, *AMI* acute myocardial infarction, *CAD* coronary artery disease, *CVA* cerebrovascular accident, *AMI* acute myocardial infarction, *PCI* percutaneous coronary intervention, *GFR* glomerular filtration rate, *MDRD* modification of diet in renal disease, *LVEF* left ventricle ejection fraction, *p-PCI* primary PCI
